# Neuropathy following spinal nerve injury shares features with the irritable nociceptor phenotype: A back‐translational study of oxcarbazepine

**DOI:** 10.1002/ejp.1300

**Published:** 2018-08-28

**Authors:** Ryan Patel, Mateusz Kucharczyk, Carlota Montagut‐Bordas, Stevie Lockwood, Anthony H. Dickenson

**Affiliations:** ^1^ Department of Neuroscience, Physiology and Pharmacology University College London London UK

## Abstract

**Background:**

The term ‘irritable nociceptor’ was coined to describe neuropathic patients characterized by evoked hypersensitivity and preservation of primary afferent fibres. Oxcarbazepine is largely ineffectual in an overall patient population, but has clear efficacy in a subgroup with the irritable nociceptor profile. We examine whether neuropathy in rats induced by spinal nerve injury shares overlapping pharmacological sensitivity with the irritable nociceptor phenotype using drugs that target sodium channels.

**Methods:**

*In vivo* electrophysiology was performed in anaesthetized spinal nerve ligated (SNL) and sham‐operated rats to record from wide dynamic range (WDR) neurones in the ventral posterolateral thalamus (VPL) and dorsal horn.

**Results:**

In neuropathic rats, spontaneous activity in the VPL was substantially attenuated by spinal lidocaine, an effect that was absent in sham rats. The former measure was in part dependent on ongoing peripheral activity as intraplantar lidocaine also reduced aberrant spontaneous thalamic firing. Systemic oxcarbazepine had no effect on wind‐up of dorsal horn neurones in sham and SNL rats. However, in SNL rats, oxcarbazepine markedly inhibited punctate mechanical‐, dynamic brush‐ and cold‐evoked neuronal responses in the VPL and dorsal horn, with minimal effects on heat‐evoked responses. In addition, oxcarbazepine inhibited spontaneous activity in the VPL. Intraplantar injection of the active metabolite licarbazepine replicated the effects of systemic oxcarbazepine, supporting a peripheral locus of action.

**Conclusions:**

We provide evidence that ongoing activity in primary afferent fibres drives spontaneous thalamic firing after spinal nerve injury and that oxcarbazepine through a peripheral mechanism exhibits modality‐selective inhibitory effects on sensory neuronal processing.

**Significance:**

The inhibitory effects of lidocaine and oxcarbazepine in this rat model of neuropathy resemble the clinical observations in the irritable nociceptor patient subgroup and support a mechanism‐based rationale for bench‐to‐bedside translation when screening novel drugs.

## 1 INTRODUCTION

Neuropathic pain remains poorly treated for the majority of patients. Emerging evidence, largely from *post hoc* analysis of clinical trial data, supports that sensory profiling of neuropathic patients could improve patient outcomes by targeting specific underlying mechanisms (Bouhassira & Attal, 2016; Simpson et al., 2010; Yarnitsky, Granot, Nahman‐Averbuch, Khamaisi, & Granovsky, 2012). Rowbotham and Fields initially described two sensory profiles in post‐herpetic neuralgia patients. The first was largely characterized by sensory loss and ongoing pain which was insensitive to local lidocaine infusion, whereas in the second group severe allodynia was the most prominent sensory disturbance, but in these cases local lidocaine alleviated ongoing pain (Rowbotham & Fields, 1989). The latter group is more commonly described as the irritable nociceptor phenotype and the former as non‐irritable/deafferentation‐type pain, although the features of both sensory phenotypes can be present within the same patient (Fields, Rowbotham, & Baron, 1998). Each phenotype may be determined by distinct mechanisms which potentially provide unique opportunities for treatment.

Oxcarbazepine and carbamazepine are recommended as front‐line treatments for trigeminal neuralgia (Cruccu et al., 2008). In contrast, there is a paucity of reliable evidence for efficacy in neuropathic patients with meta‐analyses concluding minimal/no benefit compared to placebo (Wiffen, Derry, Moore, & Kalso, 2014; Zhou et al., 2017). In pre‐clinical models, the reported efficacy of carbamazepine, and structurally related drugs, is variable and may relate to the nature of the injury and/or endpoints used in these investigations (Chapman, Suzuki, Chamarette, Rygh, & Dickenson, 1998; Fox, Gentry, Patel, Kesingland, & Bevan, 2003; Hunter et al., 1997; Jang, Kim, Park, Lee, & Moon, 2005; Lau et al., 2013). In this study, we aimed to back‐translate two clinical observations based on sensory profiling providing preliminary evidence that patients with the irritable nociceptor phenotype may benefit from the anticonvulsant oxcarbazepine (Demant et al., 2014) and a topical lidocaine patch against certain secondary pain measures (Demant et al., 2015). To this end, we examined the effects of lidocaine and oxcarbazepine in the spinal nerve ligation (SNL) model, which involves selective ligation and injury of the L5 and L6 spinal nerves, and preservation of L4 afferents.

In vivo electrophysiology was performed to determine drug effects on sensory neuronal processing at the spinal and thalamic levels. The lateral thalamic projection pathway is crucial to the sensory‐discriminative dimension of pain and is supported by electrophysiological and micro‐stimulation studies in patients undergoing neurosurgical procedures (Davis et al., 1999; Lee, Dougherty, Antezana, & Lenz, 1999; Lenz et al., 1993; Ohara, Weiss, & Lenz, 2004). The fine‐tuned coding of spinothalamic wide dynamic range (WDR) neurones accurately relates to the ability to detect small differences in the intensity of noxious stimuli (Dubner, Kenshalo, Maixner, Bushnell, & Oliveras, 1989; Maixner, Dubner, Bushnell, Kenshalo, & Oliveras, 1986), and following peripheral nerve injury in rats WDR neurones in the VPL can exhibit evoked hyperexcitability and elevated ongoing activity (Patel & Dickenson, 2016). Importantly, in parallel human and rodent studies, the relationship between rat spinal WDR properties and human pain thresholds has also been described (Coghill, Mayer, & Price, 1993; O'Neill, Sikandar, McMahon, & Dickenson, 2015; Sikandar, Ronga, Iannetti, & Dickenson, 2013). Hence, these characterizations represent an objective and quantifiable neural substrate of sensory function that overcomes many of the limitations of reflexive endpoints.

## 2 METHODS

### 2.1 Animals

Sham or spinal nerve ligated (14–18 days post‐surgery) male Sprague‐Dawley rats (250–300 g) were used for electrophysiological experiments (Biological Services, University College London, UK). Animals were group‐housed (maximum of 4) on a conventional 12:12‐hr light–dark cycle; food and water were available ad libitum. Temperature (20–22°C) and humidity (55%–65%) of holding rooms were closely regulated. All procedures described here were approved by the UK Home Office, adhered to the Animals (Scientific Procedures) Act 1986 and were designed in accordance with ethics guidelines outlined by the International Association for the Study of Pain (Zimmermann, 1983).

### 2.2 Spinal nerve ligation (SNL) surgery

Spinal nerve ligation surgery was performed as previously described (Ho Kim & Mo Chung, 1992). Rats (130–140 g) were maintained under 2% v/v isoflurane anaesthesia delivered in a 3:2 ratio of nitrous oxide and oxygen. Under aseptic conditions a paraspinal incision was made and the tail muscle excised. Part of the L5 transverse process was removed to expose the left L5 and L6 spinal nerves, which were then isolated with a glass nerve hook (Ski‐Ry, London, UK) and ligated with a non‐absorbable 6–0 braided silk thread proximal to the formation of the sciatic nerve. The surrounding skin and muscle was closed with absorbable 4–0 sutures. Sham surgery was performed in an identical manner omitting the nerve isolation and ligation step. All rats groomed normally and gained weight in the following days post‐surgery.

### 2.3 In vivo electrophysiology

#### 2.3.1 Thalamic recordings

Rats were initially anaesthetized with 3.5% v/v isoflurane delivered in a 3:2 ratio of nitrous oxide and oxygen. Once areflexic, a tracheotomy was performed and rats were subsequently maintained on 1.5% v/v isoflurane for the remainder of the experiment (approximately 3–4 hr). Core body temperature was maintained with the use of a homeothermic blanket, and respiratory rate was visually monitored throughout. Rats were secured in a stereotaxic frame, and after the was the skull exposed, coordinates for the right ventral posterolateral nucleus (VPL) of the thalamus (contralateral to injury) were calculated in relation to bregma (2.28 mm caudal, 3.2 mm lateral) (Watson & Paxinos, 2006). A small craniotomy was performed with a high‐speed surgical micro‐drill. For spinal lidocaine experiments, a partial laminectomy was performed to expose the L4‐L6 lumbar region and the overlying dura was removed. Extracellular recordings were made from VPL thalamic neurones with receptive fields on the glabrous skin of the left paw hind toes (Supporting Information Figure S1 for stereotaxically determined recording sites) using 127‐μm‐diameter 2‐MΩ parylene‐coated tungsten electrodes (A‐M Systems, Sequim, WA). Searching involved light tapping of the hind paw. Neurones were classified as wide dynamic range (WDR) on the basis of neuronal sensitivity to dynamic brushing (i.e. gentle stroking with a squirrel‐hair brush), and noxious punctate mechanical (60 g) and heat (48°C) stimulation of the receptive field. The receptive field was then stimulated using a range of natural stimuli (brush, von Frey filaments – 2, 8, 15, 26 and 60 g and heat – 35, 42, 45 and 48°C) applied over a period of 10 s per stimulus. The heat stimulus was applied with a constant water jet onto the centre of the receptive field. Acetone and ethyl chloride (100 μl) were applied as an evaporative innocuous cooling and noxious cooling stimulus, respectively (Leith, Koutsikou, Lumb, & Apps, 2010), and responses quantified over 10 s post‐application. Evoked responses to room temperature water (25°C) were minimal, or frequently completely absent, and subtracted from acetone and ethyl chloride evoked responses to control for any concomitant mechanical stimulation during application. Stimuli were applied starting with the lowest intensity stimulus with approximately 30–40 s between stimuli in the following order: brush, von Frey, cold and heat.

For lidocaine experiments, lidocaine hydrochloride (Sigma, UK) was either applied directly to the dorsal aspect of the spinal cord (20 μl, 10% w/v), or injected subcutaneously into the receptive field (20 μl, 4% w/v). Spontaneous neuronal activity was quantified over a period of 60 s immediately prior to delivery of lidocaine and again once complete block of evoked firing was confirmed. For oxcarbazepine experiments, after obtaining baseline evoked responses (mean of three trials), oxcarbazepine (Sigma, UK) was injected subcutaneously into the right flank (30 mg/kg, 1 ml/kg, vehicle: 85% normal saline, 10% Cremophor, 5% DMSO) and neuronal responses to natural stimuli were tested at 10, 30 and 50 min post‐dosing. The drug dose was guided by a previous study (Jang et al., 2005), and the effectiveness was confirmed in a pilot experiment. For licarbazepine studies, licarbazepine (Tocris, Abingdon, UK) was injected subcutaneously into the receptive field (100 μg/20 μl, vehicle: 99% normal saline, 1% DMSO) and neuronal responses to natural stimuli were tested at 5 and 25 min post‐dosing; time point of peak change is plotted for both spontaneous and evoked measures. An effective dose was determined on the basis of a pilot experiment. For all intraplantar injections, the needle was inserted adjacent to the receptive field to avoid direct tissue damage.

Data were captured and analysed by a CED1401 interface coupled to a computer with Spike2 v4 software (Cambridge Electronic Design, Cambridge, United Kingdom). The signal was amplified (×6000), band‐pass‐filtered (low‐/high‐frequency cut‐off 1.5/2 kHz) and digitized at rate of 20 kHz. Spike sorting was performed *post hoc* with Spike2 using fast Fourier transform followed by three‐dimensional principal component analysis of waveform feature measurements for multi‐unit discrimination. Neurones were recorded from one site per rat; one to three neurones were characterized at each site. Stimulus‐evoked neuronal responses were determined by subtracting total spontaneous neuronal activity in the 10‐s period immediately preceding stimulation. Spontaneous neuronal activity was measured as follows. The single spike firing rate of individual neurones (i.e. all stimulus‐independent neuronal events, including those in bursts, expressed as mean number of spikes per second) and the burst firing rate (number of bursts per second) were determined over a period of 60 s in the absence of stimulation as previously described (Hains, Saab, & Waxman, 2006). Neuronal events were considered as bursts based on the following parameters: maximum initial interval signifying burst onset (6 ms), longest interspike interval allowed within burst (9 ms) and minimum number of events in a burst (2).

#### 2.3.2 Dorsal horn recordings

Rats were anaesthetized as described above (2.3.1) and secured in a stereotaxic frame, and a laminectomy was performed to expose L4‐L6 segments of the spinal cord. Extracellular recordings were made from deep dorsal horn WDR lamina V/VI neurones with receptive fields on the glabrous skin of the left hind toes. Non‐spontaneously active neurones were selected to allow accurate determination and separation of electrically evoked spikes. The neuronal depth, from the surface of the spinal cord, was determined stereotaxically. All recordings were made at depths delineating the deep dorsal horn laminae (Watson, Paxinos, Kayalioglu, & Heise, 2009). For all spinal experiments, one neurone was recorded per rat.

Electrical stimulation of WDR neurones was delivered transcutaneously via needles inserted into the receptive field. A train of 16 electrical stimuli (2‐ms pulses, 0.5 Hz) was applied at three times the threshold current for C‐fibre activation. Responses evoked by Aβ‐ (0–20 ms), Aδ‐ (20–90 ms) and C‐fibres (90–300 ms) were separated and quantified on the basis of latency. Neuronal responses occurring after the C‐fibre latency band were classed as post‐discharge (PD). The input (I) and the wind‐up (WU) were calculated as follows: Input = (action potentials evoked by first pulse) × total number of pulses (16); wind‐up = (total action potentials after 16 train stimulus) ‐ Input. The receptive field was also stimulated using a range of natural stimuli (see section 2.3.1). After three consecutive stable baseline responses to electrical and natural stimuli (<10% variation, data were averaged to give control values), 30 mg/kg oxcarbazepine (Sigma, UK) or vehicle (85% normal saline, 10% Cremophor, 5% DMSO) were injected subcutaneously into the right flank. Neuronal responses to electrical and natural stimuli were tested at 10, 30 and 50 min post‐dosing; time point of peak change is plotted. A total of 5 naïve, 12 sham and 33 SNL rats were used in this study. All electrophysiological studies were non‐recovery; after the last post‐drug time point, rats were terminally anaesthetized with isoflurane followed by cervical dislocation.

### 2.4 Statistics

Statistical analyses were performed using SPSS, version 25 (IBM, Armonk, NY). Heat coding and mechanical coding of neurones were compared with a two‐way repeated‐measures (RM) ANOVA, followed by a Bonferroni *post hoc* test for paired comparisons. Where appropriate, sphericity was tested using Mauchly's test; the Greenhouse–Geisser correction was applied if violated. Cold, brush and single spike/burst firings were compared with a two‐tailed paired Student's *t*‐test. Group sizes were determined by a priori calculations (α 0.05, 1‐β 0.8). All data represent mean ± 95% confidence interval (CI). *p < 0.05, **p < 0.01, ***p < 0 .001.

## 3 RESULTS

### 3.1 Systemic oxcarbazepine inhibits spinal neuronal responses in SNL but not sham rats

**Figure 1:**
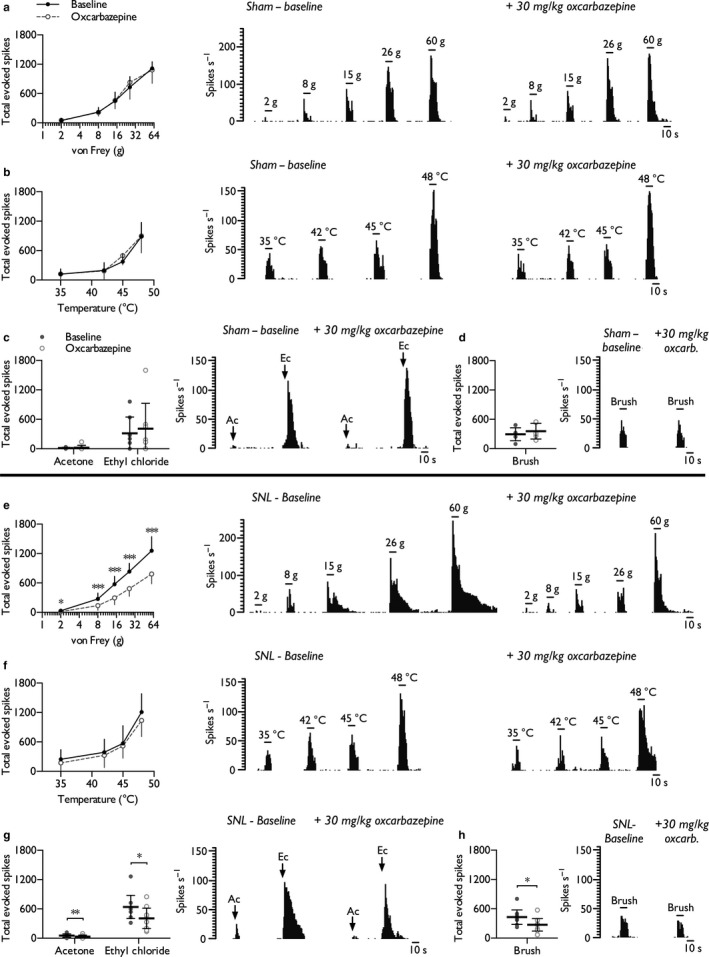
Oxcarbazepine selectively inhibits spinal neuronal responses in SNL rats in a modality‐dependent manner. Lamina V/VI WDR neuronal responses to punctate mechanical (a), heat (b), cold (c) and dynamic brush (d) stimuli prior to and following systemic administration of oxcarbazepine (30 mg/kg) in sham rats (*n *=* *7). Neuronal responses to punctate mechanical (e), heat (f), cold (g) and dynamic brush (h) stimuli prior to and following systemic administration of oxcarbazepine in SNL rats (*n *=* *8). Histogram traces represent typical single‐unit responses. Data represent mean ± 95% CI. Asterisks (*) denote difference from baseline; *p <* *0.05, **p <* *0.01, ***p <* *0.001. (Ac, acetone; Ec, ethyl chloride)

In vivo electrophysiology was performed in the dorsal horn to validate the inhibitory effect of oxcarbazepine in the SNL model. After stable baselines were obtained, sham and SNL rats were dosed subcutaneously with 30 mg/kg oxcarbazepine. In sham‐operated rats, oxcarbazepine displayed no inhibitory effect on punctate mechanical‐ (Figure 1a) (two‐way RM ANOVA *F*_1,6_ = 0.58, *p* = 0.818), heat‐ (Figure 1b) (two‐way RM ANOVA *F*_1,6_ = 0.48, *p* = 0.514), evaporative cooling‐ (Figure 1c) (paired Student's *t*‐test, acetone: *p* = 0.732; ethyl chloride: *p* = 0.418) or dynamic brush‐evoked firing (Figure 1d) (paired Student's *t*‐test, *p* = 0.143). In contrast, a marked inhibitory effect was observed in SNL rats. We found strong evidence for reduced neuronal responses to low‐intensity (2 and 8 g) and noxious (15, 26 and 60 g) punctate mechanical stimuli post‐dosing (Figure 1e) (two‐way RM ANOVA *F*_1,7_ = 112.637, *p* = 0.000014). However, only weak evidence was found for a reduction in heat‐evoked responses (Figure 1f) (two‐way RM ANOVA *F*_1,7_ = 7.362, *p* = 0.0301, paired comparisons *p* > 0.05). Both innocuous (acetone) and noxious (ethyl chloride) evaporative cooling stimuli evoked fewer spikes post‐dosing (Figure 1g) (paired Student's *t*‐test, acetone: *p *=* *0.0072; ethyl chloride: *p *=* *0.0103). In addition, oxcarbazepine inhibited neuronal responses to dynamic brushing of the receptive field (Figure 1h) (paired Student's *t*‐test, *p *=* *0.0118).

**Figure 2:**
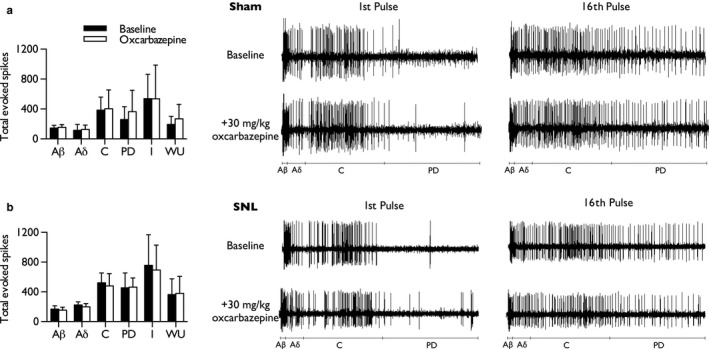
Oxcarbazepine does not inhibit wind‐up of lamina V/VI WDR neurones in either sham or SNL rats. Total neuronal events, separated according to latency, following repeated electrical stimulation in sham (a) and SNL rats (b), prior to and following systemic administration of oxcarbazepine (30 mg/kg); input and wind‐up calculated as described in Materials and Methods. Waveform traces represent typical single‐unit responses. Data represent mean ± 95% CI; sham: *n *=* *7, SNL: *n *=* *8. (PD, post‐discharge; I, input, WU, wind‐up)

Neuronal responses to repetitive stimulation were also quantified. After a sequence of supra‐threshold electrical stimuli, the total number of action potentials attributed to Aβ‐, Aδ‐, and C‐fibres did not differ from baseline in either sham (Figure 2a) (paired Student's *t*‐test: Aβ‐fibres, *p *=* *0.059; Aδ‐fibres, *p *=* *0.461; C‐fibres, *p *=* *0.768) or SNL rats (Figure 2b) (paired Student's *t*‐test: Aβ‐fibres, *p *= 0.307; Aδ‐fibres, *p *=* *0.077; C‐fibres, *p *=* *0.383). Measures of neuronal excitability, input (the non‐potentiated response), wind‐up (potentiated response) and post‐discharge were also unaffected in both sham (paired Student's *t*‐test: input, *p *=* *0.983; wind‐up, *p *=* *0.453; post‐discharge, *p *=* *0.297) (Figure 2a) and SNL rats (paired Student's *t*‐test: input, *p *=* *0.628; wind‐up, *p *=* *0.843; post‐discharge, *p *=* *0.863) (Figure 2b). The vehicle alone had no effect on either stimulus‐evoked or electrically evoked neuronal responses in naïve rats (Supporting Information Figure S2).

### 3.2 Aberrant spontaneous neuronal activity in the VPL of neuropathic rats is driven by peripheral and spinal events

**Figure 3:**
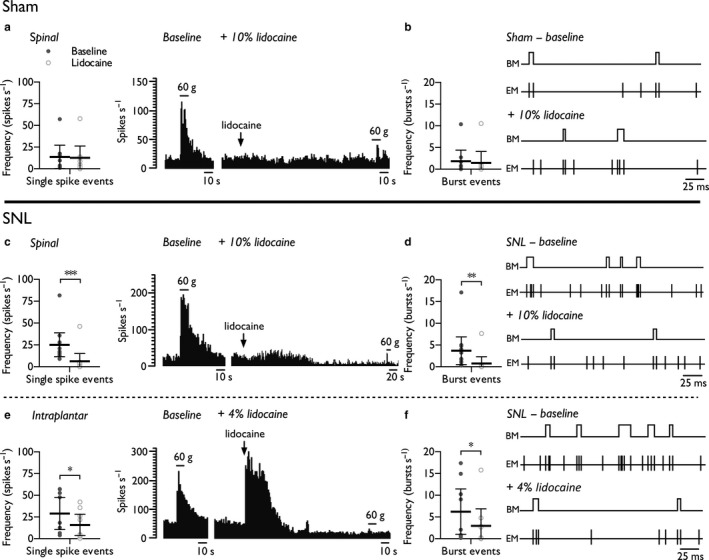
Spinal lidocaine and intraplantar lidocaine selectively attenuate spontaneous firing of thalamic WDR neurones in SNL rats. Single spike (a) and burst (b) firing rates in sham rats (*n* = 9 neurones from 5 rats) prior to and post‐spinal delivery of lidocaine (10% w/v, 20 μl). Single spike (c) and burst (d) firing rates in SNL rats (*n* = 11 neurones from 6 rats) prior to and post‐spinal delivery of lidocaine (10% w/v, 20 μl). Single spike (e) and burst (f) firing rates in SNL rats (*n* = 9 neurones from 5 rats) prior to and post‐intraplantar injection of lidocaine (4% w/v, 20 μl). Histogram and event mark traces represent typical single‐unit responses. Event markers (EM) indicate the time point of spike upstroke, and burst markers (BM) signify individual burst events. Data represent mean ± 95% CI. Asterisks (*) denote difference from baseline; *p <* *0.05, **p <* *0.01, ***p <* *0.001

We next examined integrative sensory processing in ascending pathways receiving convergent spinal output. Baseline evoked responses and spontaneous activity (Supporting Information Figure S3) were comparable to our previous observations (Patel & Dickenson, 2016; Patel, Qu, Xie, Porreca, & Dickenson, 2018). Spinally delivered lidocaine induced a complete block of evoked activity (tested with 60 g von Frey). Following confirmation of spinal block, in sham‐operated rats, the single spike firing rate (Figure 3a) (paired Student's *t*‐test, *p *=* *0.441) and burst firing rate (Figure 3b) (paired Student's *t*‐test, *p *=* *0.155) were unaffected demonstrating the absence of tonic spinal activity converging onto thalamic WDR neurones under normal conditions. In contrast, in SNL rats, spinal lidocaine substantially reduced the single spike firing rate (Figure 3c) (paired Student's *t*‐test, *p *=* *0.0001) and burst firing rate (Figure 3d) (paired Student's *t*‐test, *p *=* *0.0053) in all characterized neurones. Furthermore, we found evidence that local block of the receptive field also reduced the single spike firing rate (Figure 3e) (paired Student's *t*‐test, *p *=* *0.0123) and burst firing rate of WDR neurones (Figure 3f) (paired Student's *t*‐test, *p *=* *0.0445), although notably the effect size was greater with the spinal route of dosing for both these measures of spontaneous activity (single spike rate – spinal: Cohen's *d *=* *1.10, intraplantar: Cohen's *d *=* *0.64; burst rate – spinal: Cohen's *d *=* *0.79, intraplantar: Cohen's *d *=* *0.54).

### 3.3 Oxcarbazepine inhibits spontaneous and stimulus‐evoked neuronal activity in the VPL of neuropathic rats acting via a peripheral mechanism

**Figure 4:**
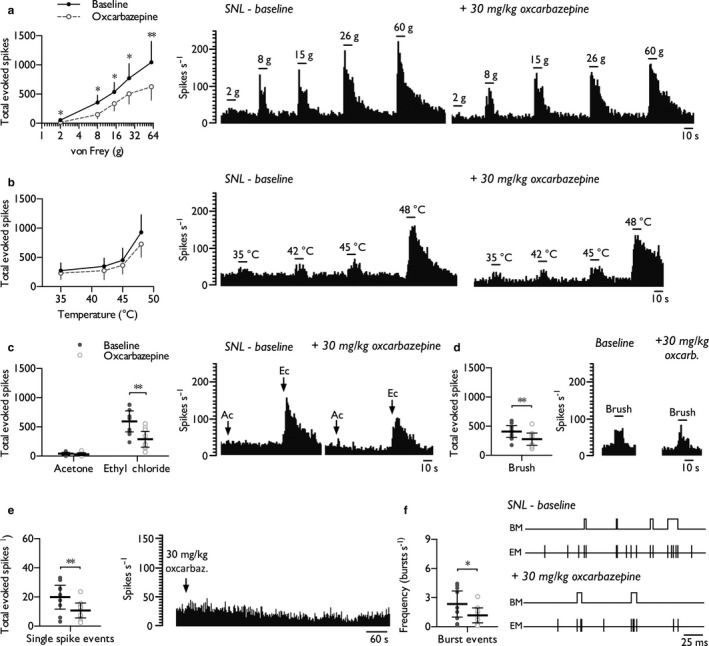
Oxcarbazepine inhibits stimulus‐evoked and spontaneous firing of thalamic WDR neurones in SNL rats. Neuronal responses to punctate mechanical (a), heat (b), cold (c), dynamic brush (d) stimuli, and spontaneous single spike (e) and burst (f) firing rates, prior to and following systemic administration of oxcarbazepine (30 mg/kg). Histogram and event mark traces represent typical single‐unit responses. Event markers (EM) indicate the time point of spike upstroke, and burst markers (BM) signify individual burst events. Data represent mean ± 95% CI, *n *=* *9 neurones from 7 rats. Asterisks (*) denote difference from baseline; *p <* *0.05, **p <* *0.01. (Ac, acetone; Ec, ethyl chloride)

The inhibitory effects of oxcarbazepine on stimulus‐evoked thalamic neuronal responses in SNL rats were comparable to the effects observed on spinal neurones. Systemic oxcarbazepine (30 mg/kg) inhibited neuronal responses to punctate mechanical stimuli (Figure 4a) (two‐way RM ANOVA *F*_1,8_ = 12.413, *p *=* *0.0078), had no effect on heat‐evoked responses (Figure 4b) (two‐way RM ANOVA *F*_1,8_ = 2.96, *p *= 0.124) and inhibited noxious cold‐evoked (ethyl chloride) responses (Figure 4c) (paired Student's *t*‐test, acetone: *p *=* *0.407; ethyl chloride: *p *= 0.0094) and brush‐evoked responses (Figure 4d) (paired Student's *t*‐test, *p *= 0.0029). In addition, spontaneous activity, that is the single spike firing rate (Figure 4e) (paired Student's *t*‐test, *p *=* *0.0014) and burst firing rate (Figure 4f) (paired Student's *t*‐test, *p *=* *0.0117), was reduced post‐dosing in 7/9 units tested.

**Figure 5:**
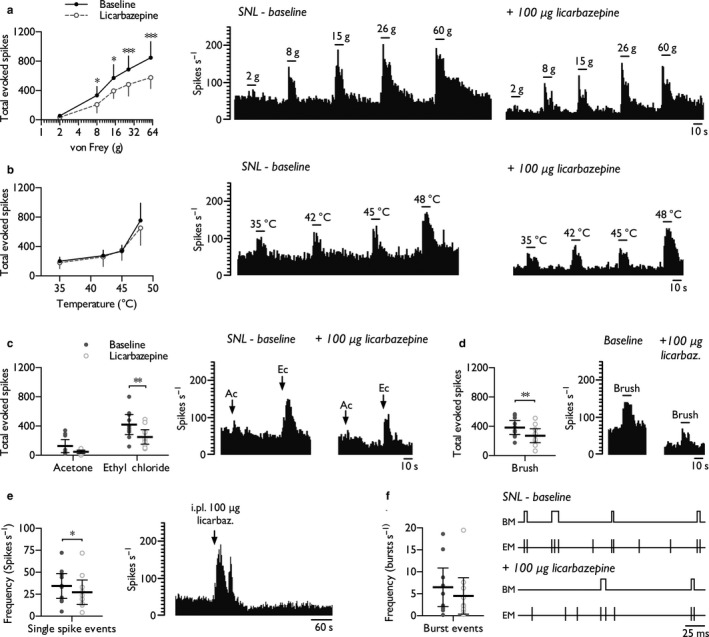
Intraplantar licarbazepine replicates the inhibitory effects of systemic oxcarbazepine on thalamic neuronal excitability in SNL rats. WDR neuronal responses to punctate mechanical (a), heat (b), cold (c), dynamic brush (d) stimuli, and spontaneous single spike (e) and burst (f) firing rates, prior to and following intraplantar injection of licarbazepine (100 μg/20 μl). Histogram and event mark traces represent typical single‐unit responses. Event markers (EM) indicate the time point of spike upstroke, and burst markers (BM) signify individual burst events. Data represent mean ± 95% CI, *n *=* *10 neurones from 7 rats. Asterisks (*) denote difference from baseline; *p <* *0.05, ***p <* *0.001. (Ac, acetone; Ec, ethyl chloride)

As oxcarbazepine is a pro‐drug that undergoes hepatic conversion, we next examined the effects of injecting the active metabolite, licarbazepine, directly into the receptive field of SNL rats to identify any peripheral mechanisms of action. Compared to systemic oxcarbazepine, intraplantar licarbazepine (100 μg/20 μl) exerted identical modality‐selective inhibitory effects. Licarbazepine inhibited neuronal responses to punctate mechanical stimuli (Figure 5a) (two‐way RM ANOVA *F*_1,9_ = 25.887, *p *=* *0.00066) and was without effect on heat‐evoked responses (Figure 5b) (two‐way RM ANOVA *F*_1,8_ = 1.23, *p *=* *0.296). Licarbazepine reduced noxious cold‐evoked responses (ethyl chloride), and weak evidence was found for similar effects in response to innocuous evaporative cooling (acetone) (Figure 5c) (paired Student's *t*‐test, acetone: *p *=* *0.06; ethyl chloride: *p *=* *0.00186). In addition, dynamic brush‐evoked neuronal responses were inhibited (Figure 5d) (paired Student's *t*‐test, *p *=* *0.0059). Licarbazepine attenuated the single spike firing rate in 7/10 units tested (Figure 5e) (paired Student's *t*‐test, *p *=* *0.0102), but only weak evidence was found for a decrease in the burst firing rate (Figure 5f) (paired Student's *t*‐test, *p *=* *0.0531). No drug effects were observed on the number of spikes per burst, burst interspike interval or burst length (Supporting Information Table S1).

## 4 DISCUSSION

Herein we describe the pathophysiological state‐dependent and modality‐selective effects of the anticonvulsant drug oxcarbazepine on stimulus‐evoked and stimulus‐independent central sensory neuronal excitability in vivo. To our knowledge, this study for the first time reports that elevated spontaneous thalamic firing in the SNL model is dependent on ongoing peripheral activity.

Oxcarbazepine is a keto analogue of carbamazepine; both are generally considered to act in a similar manner primarily as non‐selective blockers of sodium channels, but actions on potassium and calcium channels have also been reported (Ambrosio, Soares‐Da‐Silva, Carvalho, & Carvalho, 2002). Primary afferent fibres can express the TTX‐S channels Na_v_1.1, 1.6 and 1.7, and TTX‐R channels Na_v_1.8 and 1.9 (Dib‐Hajj, Cummins, Black, & Waxman, 2010); both TTX‐S and TTX‐R currents are sensitive to inhibition by carbamazepine in vitro (Rush & Elliott, 1997). These actions are via binding to inactivated sodium channels, slowing recovery into the resting state and thereby limiting channels available for opening (Hebeisen et al., 2015). Like lacosamide, carbamazepine binds to slow inactivated channels and this activation state may be associated with prolonged depolarizations that can occur in epilepsy and neuropathic pain (Cardenas, Cardenas, de Armendi, & Scroggs, 2006; Sheets, Heers, Stoehr, & Cummins, 2008). The inhibitory effects of carbamazepine are enhanced at depolarized holding potentials (Kuo, Chen, Lu, & Chen, 1997), and high‐frequency firing superimposed onto the background of altered membrane excitability may be favoured in the irritable nociceptor phenotype and trigeminal neuralgia. Intriguingly, the V400M gain‐of‐function point mutation of Na_v_1.7 that causes erythromelalgia can confer sensitivity to carbamazepine and provides insight into how the biophysical properties of sodium channel function could influence drug sensitivity in patients (Fischer et al., 2009).

The modality‐selective inhibitions exerted by oxcarbazepine (i.e. brush, punctate mechanical and cold) implicate a prominent role of Na_v_1.7, 1.8 and 1.9 channels in mediating these effects. Na_v_1.8 is predominantly expressed in nociceptors, but also a subset of non‐nociceptive afferents which could relay low‐intensity tactile information (Djouhri et al., 2003). Genetic deletion of Na_v_1.8 (Akopian et al., 1999; Matthews, Wood, & Dickenson, 2006), or pharmacological block (Patel, Brice, Lewis, & Dickenson, 2015), impairs behavioural and neuronal responses to punctate mechanical stimuli, but heat sensitivity is less affected. Ablating expression of Na_v_1.7 in sensory afferents produces deficits in noxious mechanical, heat and innocuous cold sensitivity (Minett, Eijkelkamp, & Wood, 2014; Minett et al., 2012). In addition, Na_v_1.8 and 1.9 are integral to noxious cold sensitivity (Lolignier et al., 2015; Zimmermann et al., 2007). Oxcarbazepine had no overall effect on Aβ‐, Aδ‐, and C‐fibre‐evoked responses of lamina V/VI neurones following electrical stimulation of the receptive field. The supra‐threshold and synchronized nature of the stimulus may be less amenable to inhibition compared to the prolonged activation at threshold levels of stimulation with natural stimuli. The absence of drug effects on post‐synaptically mediated potentiation perhaps indicates greater alterations in the biophysical properties of sodium channel function in primary afferents rendering second‐order neurones less susceptible to inhibition. The modality‐selective inhibitory effects are consistent with a pre‐synaptic mechanism (in peripheral and/or central terminals) as direct inhibition of post‐synaptic excitability by oxcarbazepine would reduce neuronal excitability to all peripheral inputs across sensory modalities and intensities. The absence of effect on wind‐up potentiation also argues against direct actions on spinal neurones.

Distinct roles for injured and uninjured afferents have been proposed in relation to evoked and spontaneous features of neuropathy in rats (Li, Dorsi, Meyer, & Belzberg, 2000; Yoon, Na, & Chung, 1996). After peripheral nerve injury both injured and neighbouring intact neurones exhibit increased membrane excitability and spontaneous activity, mediated in part by altered expression and distribution of voltage‐gated sodium channels (Black et al., 1999; Boucher et al., 2000; Decosterd, Ji, Abdi, Tate, & Woolf, 2002; Gold et al., 2003; Hains, Saab, Klein, Craner, & Waxman, 2004; Kim, Oh, Chung, & Chung, 2002; Ma et al., 2003; Wu et al., 2001; Zhao, Waxman, & Hains, 2006). Dorsal horn neurones in this study exhibited minimal spontaneous activity, consistent with previous characterizations reporting aberrant spontaneous firing in a subset of innervated neurones in SNL rats (Chapman, Suzuki, & Dickenson, 1998). An important consideration in reconciling the dorsal horn and thalamic neuronal properties is that dorsal horn neurones without peripheral receptive fields can be spontaneously active (Dalal et al., 1999; Suzuki & Dickenson, 2006), and an element of this ongoing activity could originate from the neuromas at L5 and L6. Thus, the summation of this spinal activity converges onto thalamic pathways. Local and spinal block with lidocaine reveals evidence of ongoing activity within ascending sensory projection pathways in the SNL model, and notably the inhibitory effect was greater following spinal block. However, we cannot infer from this observation alone whether this reflects noxious input to the thalamus, nor can we determine whether the effect of oxcarbazepine on this elevated activity is primarily due to actions on injured or uninjured primary afferents. Peripheral nerve block and intrathecal lidocaine can produce conditioned place preference after nerve or tissue injury, indicating relief from ongoing pain (He, Tian, Hu, Porreca, & Wang, 2012; Navratilova et al., 2012), and this could occur independently of activity within the lateral ascending pathway (Navratilova et al., 2015).

Direct evidence for actions of carbamazepine on injured nerves came from demonstrations that systemic administration blocked ectopic firing in some transected afferents (Burchiel, 1988; Yates, Smith, & Robinson, 2005). The data presented here additionally identify inhibitory drug effects on spontaneous peripheral activity distal to the injury. The systemic dose of oxcarbazepine was more effective at reducing spontaneous activity compared to the local injection of licarbazepine. This difference may arise from systemic drug actions at the level of the injury as supported by the aforementioned studies in neuromas, or by inhibiting ectopic events that originate in dorsal root ganglion neurones (Wall & Devor, 1983), or by direct actions within thalamocortical circuits. Thalamic neuronal excitability is governed by feedback from cortical regions and the thalamic reticular nucleus (TRN), which modulate ascending sensory transmission (Monconduit, Lopez‐Avila, Molat, Chalus, & Villanueva, 2006; Yen & Shaw, 2003). GABAergic inputs from the TRN can promote burst firing patterns (Cope, Hughes, & Crunelli, 2005), and the decrease in the burst firing rate observed with intraplantar lidocaine, and to some extent with intraplantar licarbazepine, is consistent with altered top‐down modulation of thalamic neuronal excitability. However, with the systemic dose of oxcarbazepine, inhibitory effects directly on thalamic neurones and/or within these modulatory circuits are also possible.

**Figure 6:**
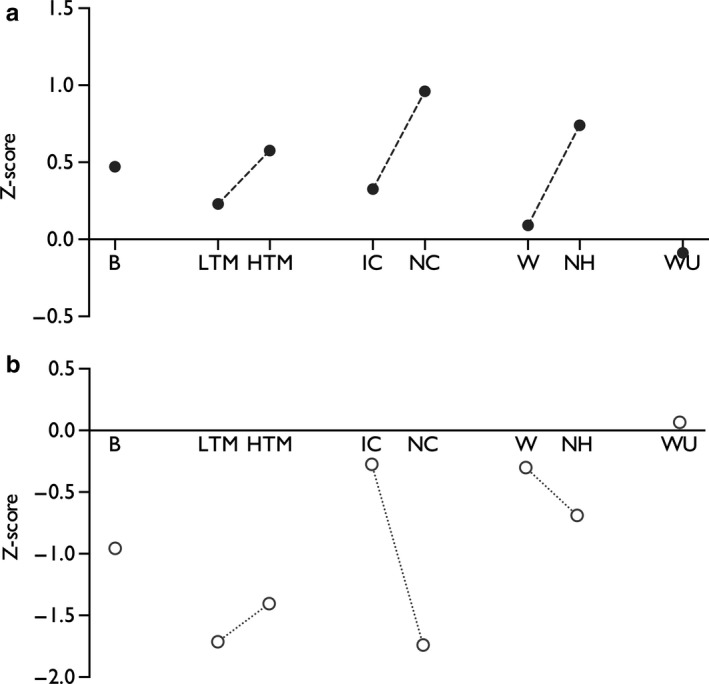
A standardized representation of sensory gain in the spinal nerve ligation model in rats (a). Historical neuronal data from the ventral posterolateral thalamus were compiled from sham/naïve (*n *=* *84 neurones from 52 rats) and SNL rats (*n *=* *111 neurones from 76 rats) to determine ‘normal’ levels of sensory coding and the normalized degree of sensory gain in SNL rats. Wind‐up was determined by collating historical data from dorsal horn recordings from sham/naïve (*n *=* *66 neurones from 66 rats) and SNL rats (*n *=* *61 neurones from 61 rats). Positive values represent gain‐of‐function, and negative values represent loss‐of‐function compared to the sham/naïve group (reproduced from Dickenson & Patel, 2018). The standardized inhibitory profile of oxcarbazepine in SNL rats (b), based on the thalamic and dorsal horn (WU‐only) neuronal responses (pre‐ and post‐dosing comparison). Positive values represent facilitation, and negative values represent inhibitory drug effects. (Measures represent: B, brush; LTM, low‐threshold punctate mechanical (2 g); HTM, high‐threshold punctate mechanical (60 g); IC, innocuous cooling (acetone), NC, noxious cooling (ethyl chloride); W, warm temperature (35°C); NH, noxious heat (48°C); WU, wind‐up. Interconnecting lines indicate modality groups)

Recently, a call has been reiterated to apply a sensory profiling approach to animal models (Baron & Dickenson, 2014; Rice, Finnerup, Kemp, Currie, & Baron, 2018); that is, models could be classified according to their sensory gain/loss akin to quantitative sensory testing (QST) in patients and drug effects should be determined against these sensory endpoints. Fundamentally, there are challenges in aligning perceptual outcomes in patients with measures in animals, but we provide an example of how a sensory profile can in part be achieved in the SNL model from thalamic neuronal recordings. Like many animal models, spinal nerve injury results in sensory gain across modalities rather than sensory loss (Figure 6A) and shares some resemblance to the ‘thermal’ phenotype (Baron et al., 2017). Baron and colleagues note that the irritable nociceptor patients are represented by the ‘thermal’ phenotype based on proposed peripheral sensitization, yet cluster 3 with mechanical hyperalgesia are suggested to have central sensitization. The sensory profile of the irritable nociceptor phenotype could result from abnormal peripheral inputs, but also from normal inputs impinging on a sensitized spinal cord. Our data indicate that SNL rats appear to overlap with both these clusters and are very different from the ‘sensory loss’ group. By normalizing the effect size, the inhibitory effect of oxcarbazepine in SNL rats is greatest against noxious cold and punctate mechanical stimuli (Figure 6b). As these are sensory modalities tested with QST, these observations could help shape translational studies and test the validity of applying a sensory profiling approach to pre‐clinical and clinical research.

These data support a reconsideration of how animal models are utilized for screening novel drugs. The homogeneity of animal models complicates the translation of pre‐clinical findings to patient groups exhibiting heterogeneous symptoms. Sensory profiles may represent a surrogate for underlying mechanisms (Baron et al., 2017; Bouhassira & Attal, 2016; Truini, Garcia‐Larrea, & Cruccu, 2013), and a mechanism‐based approach would represent a progressive way to link animal and clinical research (for example see (Yarnitsky et al., 2012) and (Bannister, Patel, Goncalves, Townson, & Dickenson, 2015)). Attempts to correlate lidocaine sensitivity to sensory profiles have not always proved successful (Haroutounian et al., 2014), although patients with the irritable nociceptor phenotype were more likely to achieve relief from a 5% lidocaine patch against pain paroxysms and deep pain (Demant et al., 2015). Intravenous lidocaine in peripheral neuropathy patients alleviated ongoing pain and mechanical allodynia without affecting thermal allodynia (Attal, Rouaud, Brasseur, Chauvin, & Bouhassira, 2004). Moreover, the degree of relief was inversely correlated with deficits in thermal sensitivity much like the early reports of the irritable nociceptor phenotype (Attal et al., 2004; Rowbotham & Fields, 1989). With regard to oxcarbazepine, NNT values (3.9 and 13, respectively) are improved in the irritable versus non‐irritable group (Demant et al., 2014). In this study, we observe that oxcarbazepine, largely ineffectual in the majority of neuropathic patients, exerts inhibitory effects in the SNL model. It is conceivable that the failure to translate the pre‐clinical effects of other state‐dependent ion channel blockers (e.g. lacosamide (Beyreuther, Callizot, & Stohr, 2006; Hearn, Derry, & Moore, 2012)) to clinical efficacy has stemmed from poor trial design that lacks stratification, and hence the sensitivity to determine positive drug effects in patient subgroups.

## AUTHOR CONTRIBUTIONS

RP and AHD conceived and designed the study; RP, MK, CMB and SL performed the experiments; RP analysed the data; RP and AHD interpreted the results of experiments; RP prepared the figures; RP drafted the manuscript; RP and AHD edited and revised the manuscript. All authors read and approved the final manuscript.

## CONFLICT OF INTEREST

The authors have no conflicts of interest to declare.
