# Supplementary material for: Neuropathy following spinal nerve injury shares features with the irritable nociceptor phenotype: A back‐translational study of oxcarbazepine
Source: Eur J Pain. 2018 Aug 28;23(1):183–97. doi: 10.1002/ejp.1300 (PMC6396087; doi:10.1002/ejp.1300)
Supplement: Supplementary file 1 [file EJP-23-183-s001.docx]

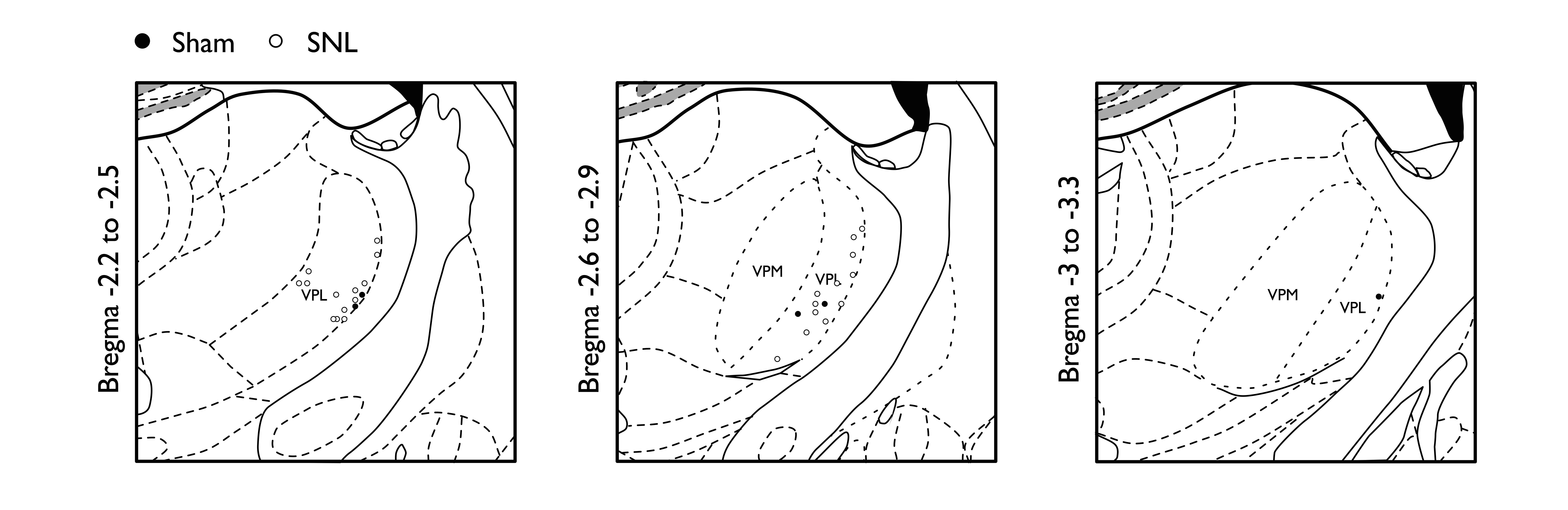


**Supplementary figure 1.** Recording sites within the ventral posterolateral (VPL) nucleus of the thalamus from 5 sham and 25 neuropathic rats. Filled circles (●) represent sham, open circles (○) represent spinal nerve ligated (SNL) rat experiments.
