# Supplementary material for: Neuropathy following spinal nerve injury shares features with the irritable nociceptor phenotype: A back‐translational study of oxcarbazepine
Source: Eur J Pain. 2018 Aug 28;23(1):183–97. doi: 10.1002/ejp.1300 (PMC6396087; doi:10.1002/ejp.1300)
Supplement: Supplementary file 2 [file EJP-23-183-s002.docx]

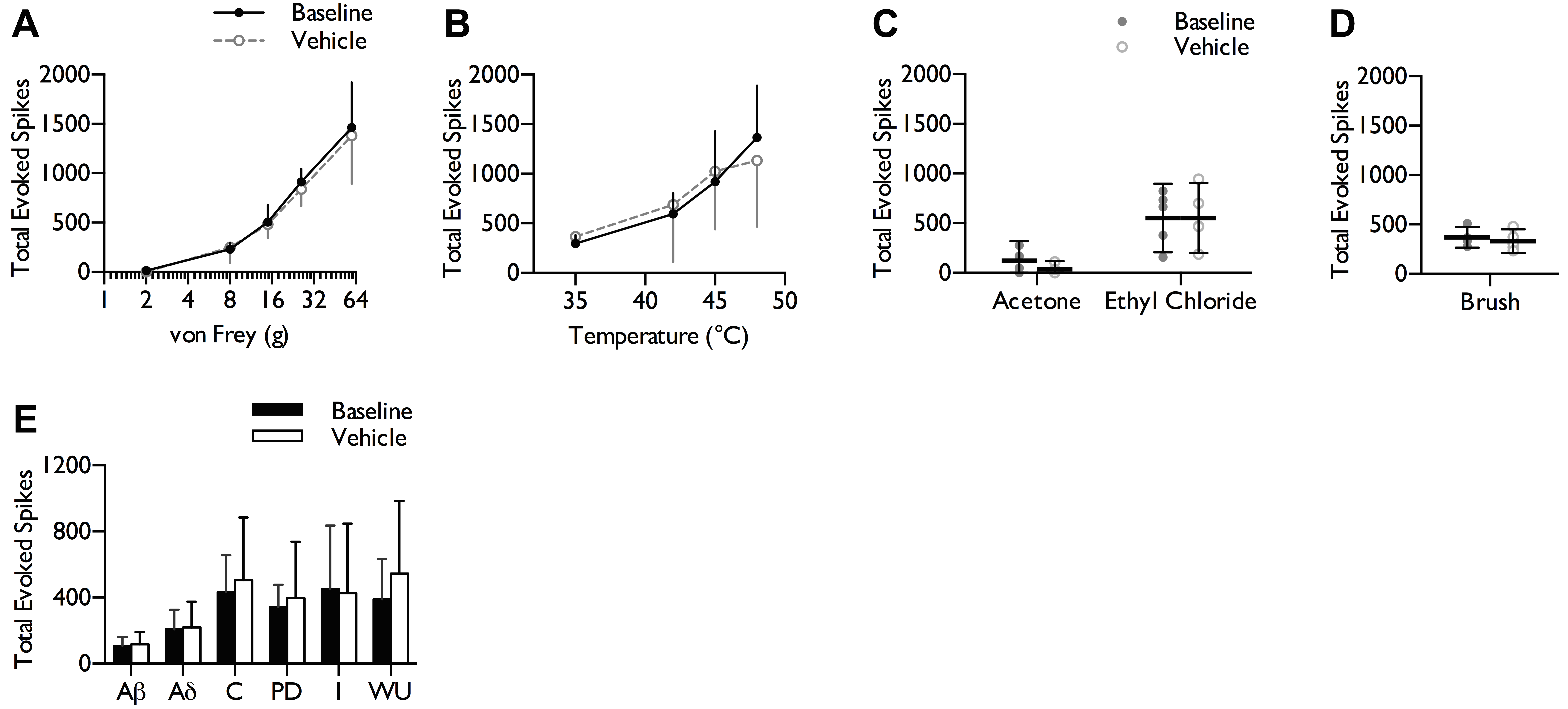


**Supplementary figure 2.** Dorsal horn lamina V/VI wide dynamic range neuronal responses to punctate mechanical (*A*), heat (*B*), evaporative cooling (*C*), dynamic brush (*D*) and electrical (*E*) stimuli, prior to and following subcutaneous injection of 1 ml/kg vehicle (85% normal saline, 10% cremophor, 5% DMSO) in naïve rats (*n*=5). Data represent mean ± 95% CI. (PD – post-discharge, I – input, WU – wind-up).
