# Supplementary material for: Neuropathy following spinal nerve injury shares features with the irritable nociceptor phenotype: A back‐translational study of oxcarbazepine
Source: Eur J Pain. 2018 Aug 28;23(1):183–97. doi: 10.1002/ejp.1300 (PMC6396087; doi:10.1002/ejp.1300)
Supplement: Supplementary file 3 [file EJP-23-183-s003.docx]

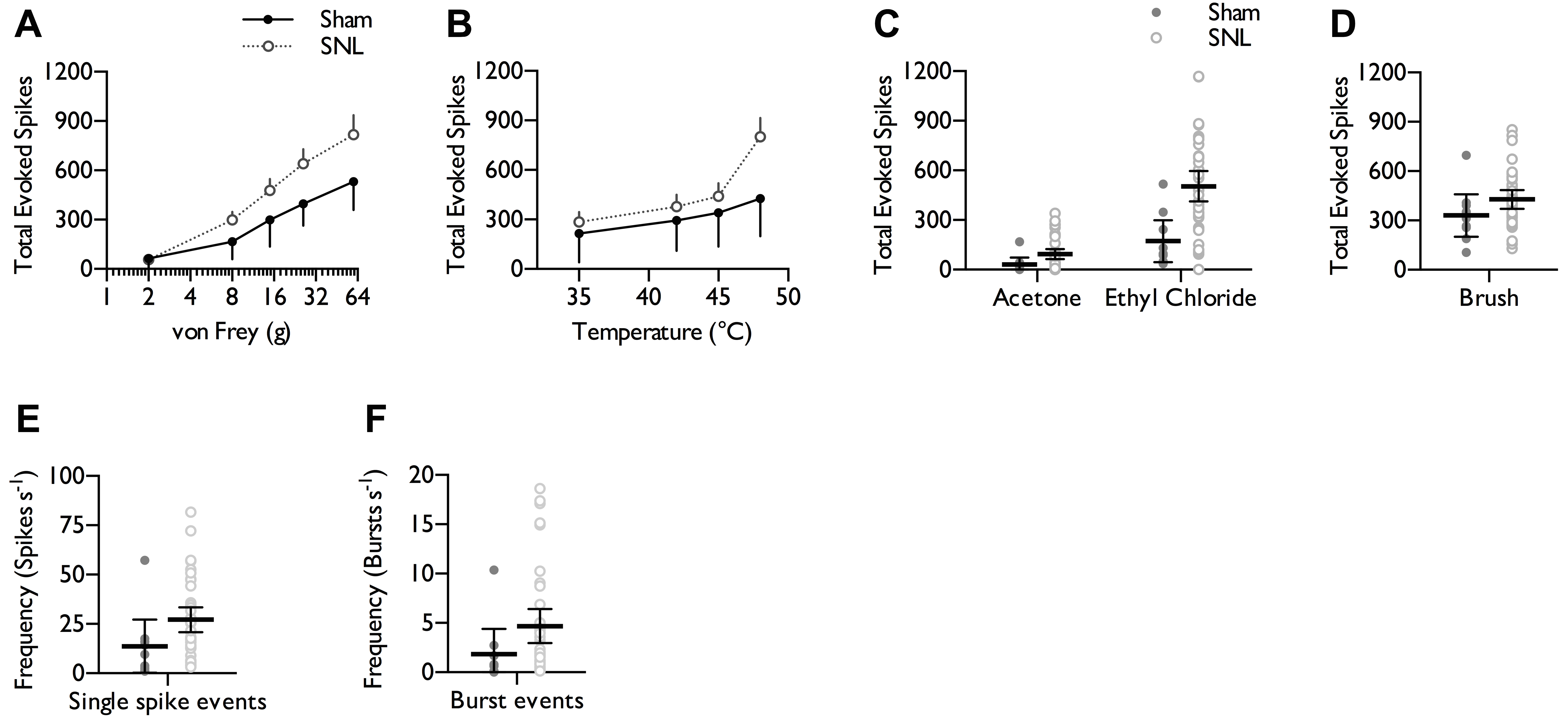


**Supplementary figure 3.** Baseline responses of wide dynamic range neurones in the ventral posterolateral thalamus to punctate mechanical (*A*), heat (*B*), evaporative cooling (*C*) and dynamic brush (*D*) stimulation of the receptive field, and spontaneous single spike (*E*) and burst (*F*) firing rates. SNL: *n*=39 neurones from 25 rats, sham: *n*=9 neurones from 5 rats. Data represent mean ± 95% CI.
