# Supplementary material for: Neuropathy following spinal nerve injury shares features with the irritable nociceptor phenotype: A back‐translational study of oxcarbazepine
Source: Eur J Pain. 2018 Aug 28;23(1):183–97. doi: 10.1002/ejp.1300 (PMC6396087; doi:10.1002/ejp.1300)
Supplement: Supplementary file 4 [file EJP-23-183-s004.docx]

|  | **SNL** | | | | | |  | **SNL** | | | | | |  | **SNL** | | | | | |  | **SNL** | | | | | |  | **Sham** | | | | | |
| --- | --- | --- | --- | --- | --- | --- | --- | --- | --- | --- | --- | --- | --- | --- | --- | --- | --- | --- | --- | --- | --- | --- | --- | --- | --- | --- | --- | --- | --- | --- | --- | --- | --- | --- |
|  | **Baseline** | | | **Oxcarbazepine** | | |  | **Baseline** | | | **Licarbazepine** | | |  | **Baseline** | | | **Spinal lidocaine** | | |  | **Baseline** | | | **I.pl lidocaine** | | |  | **Baseline** | | | **Spinal lidocaine** | | |
| **% of spikes in bursts** | 23.31 | ± | 3.98 | 19.61 | ± | 2.95 |  | 38.23 | ± | 6.53 | 30.82 | ± | 6.26 |  | 26.03 | ± | 5.14 | 10.07 | ± | 3.85 |  | 30.62 | ± | 7.82 | 15.64 | ± | 9.11 |  | 25.49 | ± | 6.92 | 16.49 | ± | 7.76 |
| **Mean spikes per burst** | 2.22 | ± | 0.14 | 2.00 | ± | 0.00 |  | 2.40 | ± | 0.16 | 2.10 | ± | 0.10 |  | 2.09 | ± | 0.09 | 2.22 | ± | 0.13 |  | 2.22 | ± | 0.14 | 2.00 | ± | 0.00 |  | 2.40 | ± | 0.22 | 2.29 | ± | 0.15 |
| **Mean burst interspike (ms)** | 1.94 | ± | 0.09 | 1.69 | ± | 0.08 |  | 1.98 | ± | 0.13 | 2.09 | ± | 0.09 |  | 1.93 | ± | 0.08 | 1.77 | ± | 0.19 |  | 1.91 | ± | 0.19 | 1.90 | ± | 0.17 |  | 1.68 | ± | 0.19 | 2.10 | ± | 0.10 |
| **Mean burst length (ms)** | 4.59 | ± | 0.42 | 3.65 | ± | 0.22 |  | 4.75 | ± | 0.45 | 4.81 | ± | 0.36 |  | 4.63 | ± | 0.39 | 4.01 | ± | 0.45 |  | 4.51 | ± | 0.60 | 4.08 | ± | 0.40 |  | 4.31 | ± | 0.72 | 5.13 | ± | 0.65 |

**Supplementary table 1.** Summary of spontaneous burst characteristics prior to and post drug delivery. Data represent mean ± 95% CI.
